# Clinical condition of 120 patients alive at 3 years after poor-grade aneurysmal subarachnoid hemorrhage

**DOI:** 10.1007/s00701-021-04725-2

**Published:** 2021-02-25

**Authors:** Anniina H. Autio, Juho Paavola, Joona Tervonen, Maarit Lång, Terhi J. Huuskonen, Jukka Huttunen, Virve Kärkkäinen, Mikael von und zu Fraunberg, Antti E. Lindgren, Timo Koivisto, Juha E. Jääskeläinen, Olli-Pekka Kämäräinen

**Affiliations:** 1 Neurosurgery of NeuroCenter, Kuopio University Hospital, PL 100, 70029 Kuopio, Finland; 2 Neurointensive Care Unit, Kuopio University Hospital, Kuopio, Finland; 3 Clinical Radiology, Kuopio University Hospital, Kuopio, Finland; 4 Institute of Clinical Medicine, School of Medicine, Faculty of Health Sciences, University of Eastern Finland, Kuopio, Finland

**Keywords:** Aneurysmal subarachnoid hemorrhage, Hunt and Hess scale 4–5, Long-term outcome, Modified Rankin Scale

## Abstract

**Background:**

To study the clinical condition of poor-grade aneurysmal subarachnoid hemorrhage (aSAH) patients alive at 3 years after neurointensive care.

**Methods:**

Of the 769 consecutive aSAH patients from a defined population (2005–2015), 269 (35%) were in poor condition on admission: 145 (54%) with H&H 4 and 124 (46%) with H&H 5. Their clinical lifelines were re-constructed from the Kuopio Intracranial Aneurysm Database and Finnish nationwide registries. Of the 269 patients, 155 (58%) were alive at 14 days, 125 (46%) at 12 months, and 120 (45%) at 3 years.

**Results:**

The 120 H&H 4–5 patients alive at 3 years form the final study population. On admission, 73% had H&H 4 but only 27% H&H 5, 59% intracerebral hematoma (ICH; median 22 cm^3^), and 26% intraventricular blood clot (IVH). The outcome was favorable (mRS 0–1) in 45% (54 patients: ICH 44%; IVH clot 31%; shunt 46%), moderate (mRS 2–3) in 30% (36 patients: ICH 64%; IVH clot 19%; shunt 42%), and unfavorable (mRS 4–5) in 25% (30 patients: ICH 80%; IVH clot 23%; shunt 50%). A total of 46% carried a ventriculoperitoneal shunt. ICH volume was a significant predictor of mRS at 3 years.

**Conclusions:**

Of poor-grade aSAH patients, 45% were alive at 3 years, even 27% of those extending to pain (H&H 5). Of the survivors, 75% were at least in moderate condition, while only 2.6% ended in hospice care. Consequently, we propose non-selected admission to neurointensive care (1) for a possibility of moderate outcome, and (2), in case of brain death, possibly improved organ donation rates.

## Introduction

Aneurysmal subarachnoid hemorrhage (aSAH), in most cases from a ruptured saccular intracranial aneurysm (sIA), is the third most frequent form of stroke [26, 50]. The mechanisms of acute brain injury include intracerebral hemorrhage (ICH), intraventricular hemorrhage (IVH), acute brain ischemia, acute hydrocephalus, increased intracranial pressure (ICP), and seizures [7, 9, 46]. Further deterioration may be due to uncontrollable ICP, delayed ischemic brain injury, electrolyte disturbances, cardiopulmonary complications, and CNS or systemic infections [7, 9, 12, 40, 46].

Poor condition on admission is usually expressed as Hunt and Hess (H&H) scale 4 or 5, or World Federation of Neurosurgical Societies (WFNS) grade 4 or 5 [6, 48]. The extent of intracranial bleeding is expressed as CT scales, including the Fisher scale, while computed bleeding volume and site analyses are emerging [13, 40]. Poor condition predicts high early mortality, and aSAH patients brain dead within 14 days are a significant group of organ donors [21]. In Finland, with national presumed consent (opt-out) since 2010 [21, 45], aSAH patients with dismal prognosis can be admitted to neurointensive care as potential organ donors, also at high age. Non-selected admission to neurointensive care would increase organ donation [21, 45]—but is long-term hospice care survival (mRS 5) increasing as well? The long-term mortality is often presented at 12 months [47]: 49% for H&H 4 and 92% for H&H 5 in our previous study [22]. A minority of H&H 4–5 or WFNS 4–5 patients will become true long-term survivors, alive e.g., at 5 years or 10 years, albeit being stroke-risk carriers and prone to further vascular events [16]. They have remained an unrecognized group among stroke survivors for obvious reasons: prospective databases with long recruitment and outpatient follow-up periods would be virtually impossible to maintain, while much of literature is focused on “aneurysm treatment” [43].

We constructed the clinical lifelines of 269 consecutive aSAH patients with H&H 4 or 5 on admission to the Neurointensive Care Unit of the Kuopio University Hospital (KUH) from a defined population between 2005 and 2015 by using the nationwide registries and personal identity codes. Our aim is to present a cross-sectional analysis of the 120 (45%) H&H 4–5 patients alive at 3 years. The 120 primary CT scans (ICH; IVH) and the interventions (ICH evacuation; decompressive craniectomy; shunt) are compiled together, according to the modified Rankin Scale (mRS) at 3 years.

## Methods and materials

### Kuopio University Hospital (KUH) and aSAH management protocol in Eastern Finland

KUH, one of the five university hospitals in Finland, is an academic, non-profit, publicly funded tertiary center, which serves a defined population (about 842,000 in 2015) in Eastern Finland. The KUH area contains four central hospitals, each with 24/7 neuroacutology, CT services, intensive care, neurology, and neurorehabilitation. The KUH area is served by ambulance and (since 2002) by helicopter transfer. At KUH Neurosurgery, at least two neurosurgeons are on duty at all times. During the study period from 2005 to 2015, all cases of SAH were acutely transferred to KUH for neurointensive care, neuroradiology (4-vessel angiography and/or CT angiography), and neurosurgery [21]. Neurointensive care was provided to virtually all patients regardless of the age or condition on admission, including H&H 4–5 patients [21]. A dedicated team of neurointensivists, neurosurgeons, and interventional neuroradiologists coordinated the aSAH treatment. The KUH Neurovascular Group provided microsurgical or endovascular occlusion of the ruptured aneurysm and evacuated significant ICHs with immediate microsurgery. The KUH aSAH neurointensive care protocol in 2005–2015 followed international recommendations in detail [9, 27, 29, 44, 46], and it was presented in our previous study of organ donors after aSAH [21, 45]. Briefly, the protocol aimed to prevent further brain damage due to rebleeding, increased intracranial pressure (ICP), hydrocephalus, electrolyte disturbances, seizures, cardiac and pulmonary dysfunction, fever, hyperglycemia, and development of delayed brain ischemia. The protocol included, when appropriate, e.g., external ventricular drainage (EVD), parenchymal ICP monitoring, endovascular procedures, and intra-arterial nimodipine infusion in case of delayed brain ischemia, as well as decompressive craniectomy (DC).

### Kuopio Intracranial Aneurysm Patient and Family Database

The database, prospective since 1995, contains all cases of unruptured and ruptured IAs admitted to KUH since 1980. A dedicated, full-time nurse administrates the database, interviews all new IA patients, including their family history, and arranges the follow-ups. The clinical data, including prescribed medicines, hospital diagnosis, and causes of death, have been fused from the national registries, using the Finnish personal codes. We have characterized the aSAH patients, e.g., for the 14-day mortality and organ donation [21], 12-month [22] and long-term excess mortality [17], shunt-dependent hydrocephalus [1], depression [19], epilepsy [18], pain [33], anti-psychotics [39], diabetes mellitus [31], hypertension [25, 32], as well as, polycystic kidney disease [36]. Three first-degree relatives with a diagnosed sIA disease form an sIA family.

### Basic study population of 269 aSAH patients in poor condition on admission

**Fig. 1:**
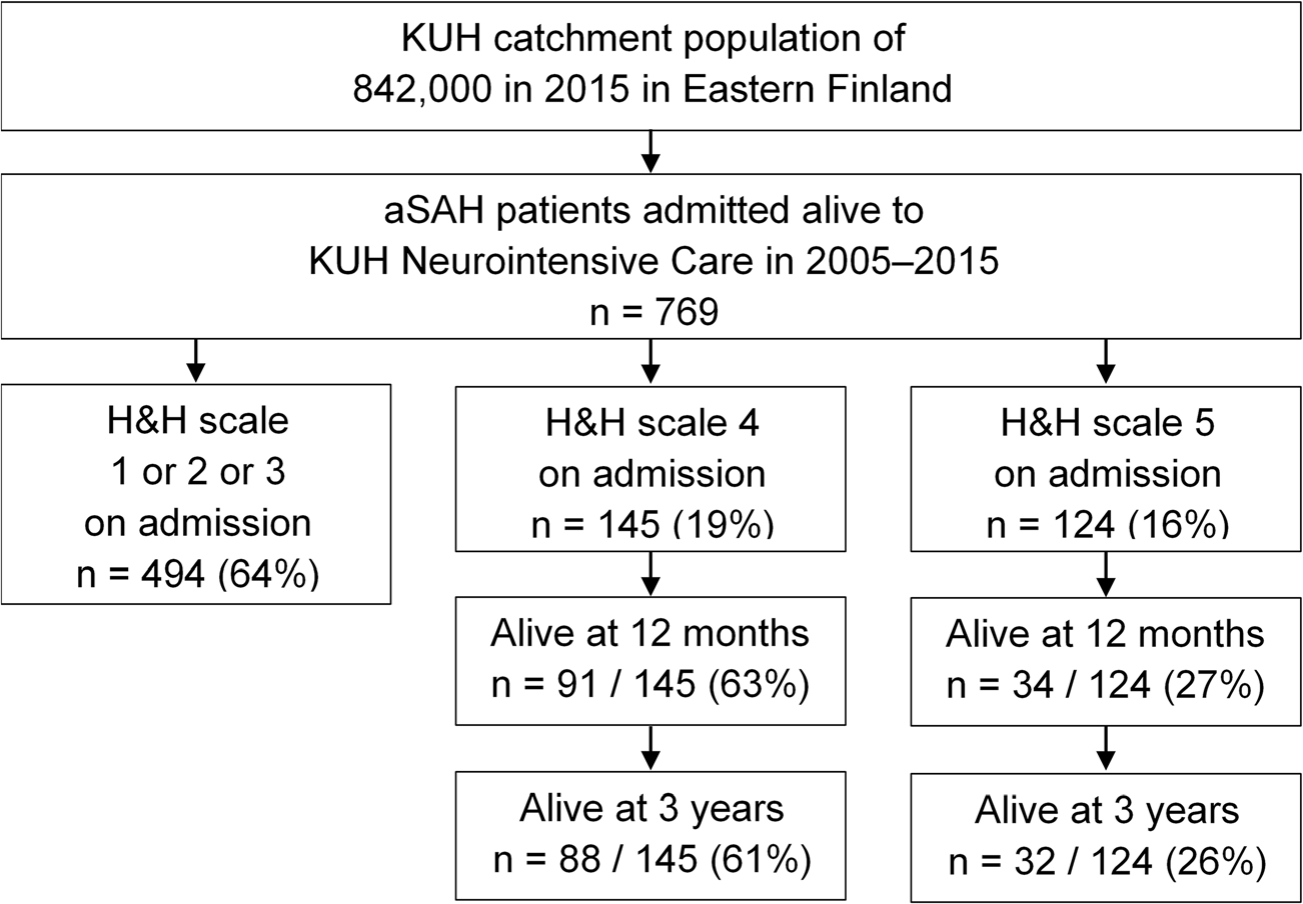
Flowchart. A total of 769 patients had been acutely admitted for aneurysmal subarachnoid hemorrhage (aSAH) to the neurosurgical and neurointensive care between 2005 and 2015 from Eastern Finnish catchment population. aSAH, aneurysmal subarachnoid hemorrhage; H&H, Hunt and Hess scale

A total of 769 consecutive aSAH patients were acutely admitted to the KUH Neurointensive Care Unit from 2005 to 2015 (Fig. 1). The six patients lost to follow-up were excluded. A total of 269 (35%) aSAH patients had H&H 4 (*n*=145) or H&H 5 (*n*=124; extension to pain) on arrival or before intubation before arrival to the KUH (Fig. 1). Their clinical lifelines were re-constructed from their clinical data in the Kuopio database and from the national clinical registries until death (*n*=149) or July 2019 (Table 1). The patients who deteriorated from H&H 1–3 on admission to H&H 4–5 during the neurointensive care were excluded.

**Table 1:** Characteristics of 269 consecutive aneurysmal subarachnoid hemorrhage (aSAH) patients admitted acutely in poor condition (H&H scale 4 or 5) to the Neurointensive Care Unit of the Kuopio University Hospital from its Eastern Finnish catchment population from 2005 to 2015. The number of patients alive at 12 months and 3 years is given. The final study population consisted of 120 patients alive at 3 years, 88 with H&H 4 and 32 with H&H 5 on admission (see Table 3)

Clinical condition on admission			H&H 4 patients			H&H 5 patients		
			On admission, *n* = 145	Alive at 12 months, *n* = 91 (63%)	Alive at 3 years, *n* = 88 (61%)	On admission, *n* = 124	Alive at 12 months, *n* = 34 (27%)	Alive at 3 years, *n* = 32 (26%)
Median age at aSAH (years) (IQR)			56 (49–64)	53 (45–62)	53 (45–61)	59 (48–66)	50 (45–60)	49 (44–60)
Females (%) Males (%)			90 (62) 55 (38)	59 (65) 32 (35)	58 (66) 30 (34)	72 (58) 52 (42)	17 (50) 17 (50)	15 (47) 17 (53)
Multiple sIAs (%)			53 (37)	33 (36)	32 (36)	28 (23)	10 (29)	10 (31)
Intracerebral hematoma (ICH) (%)			96 (66)	58 (64)	55 (63)	62 (50)	16 (47)	16 (50)
Intraventricular hematoma (IVH) Blood clot (%) Blood sediment in occipital horns of lateral ventricles (%)			41 (28) 67 (46)	22 (24) 42 (46)	22 (25) 41 (47)	51 (41) 36 (29)	11 (32) 18 (53)	9 (28) 18 (56)
Hydrocephalus on admission (%)			97 (67)	57 (63)	54 (61)	83 (67)	23 (68)	22 (69)
Extraventricular drainage (%)			127 (88)	78 (86)	76 (86)	83 (67)	31 (91)	29 (91)
Ruptured sIA/median size, mm (IQR)			7 (5–10)	7 (5–10)	7 (5–10)	8 (5–14)	7 (5–10)	7 (5–10)
Location	Total (%), *n*=269	ICH (%), *n*=158 (59)						
ICA	51 (19)	29/51 (57)	25 (17)	11 (12)	10 (11)	26 (21)	5 (15)	5 (16)
AComA	33 (12)	17/33 (52)	21 (14)	17 (19)	17 (19)	12 (10)	7 (21)	7 (22)
Other ACA	23 (9)	17/23 (74)	13 (9)	3 (3)	3 (3)	10 (8)	0 (0)	0 (0)
MCA	102 (38)	85/102 (83)	58 (40)	43 (47)	42 (48)	44 (35)	15 (44)	15 (47)
BA	44 (16)	10/44 (23)	21 (14)	14 (15)	13 (15)	23 (19)	4 (12)	2 (6)
PICA	16 (6)	0/16 (0)	7 (5)	3 (3)	3 (3)	9 (7)	3 (9)	3 (9)
Microsurgical occlusion of ruptured sIA (%)			55 (38)	41 (45)	40 (45)	25 (20)	15 (44)	15 (47)
Endovascular occlusion of ruptured sIA (%)			77 (53)	48 (53)	47 (53)	34 (27)	19 (56)	17 (53)
No sIA occlusion (%)			13 (9)	2 (2)	1 (1)	65 (52)	0 (0)	0 (0)
Decompressive craniectomy (%)			22 (15)	10 (11)	10 (11)	13 (10)	6 (18)	6 (19)
Brain dead in 2 weeks and organ donor (%)			10 (7)	n.r.	n.r.	29 (23)	n.r.	n.r.
Shunt installed (%)			43 (30)	37 (41)	35 (40)	23 (19)	22 (65)	20 (63)

*H&H*, Hunt and Hess scale; *aSAH*, aneurysmal subarachnoid hemorrhage; *IQR*, 25% and 75% range; *sIA*, saccular intracranial aneurysm; *ICH*, intracerebral hematoma; *IVH*, intraventricular hematoma; *ICA*, internal carotid artery trunk and bifurcation; *AComA*, anterior communicating artery; *ACA*, anterior cerebral artery and peripheral segments; *MCA*, middle cerebral artery and peripheral segments; *BA*, basilar artery trunk and bifurcation; *PICA*, posterior inferior cerebellar artery

### Final study population of 120 aSAH patients alive at 3 years

A total of 120 H&H 4 (*n*=88) or H&H 5 (*n*=32) patients were alive at 3 years after admission (Tables 1 and 2). Their 120 primary digital CT scans were analyzed for the presence or absence of ICH, IVH blood clot, IVH sediment, and hydrocephalus (Table 2). A representative CT slice of each patient was compiled together and sorted according to the modified Rankin Scale (mRS 0 to 5) at 3 years. The ICH volumes were calculated from the CT scans using the formula π × (*a* × *b* × *c*) / 6 where *a*, *b*, and *c* are the three perpendicular diameters of ICH. All available clinical data were obtained from the KUH electronic health care records, purchases of prescripted drugs, and all hospital and primary health care diagnoses from the national databases. By our definition, the use of anti-epileptic, anti-depressive, or anti-psychotic medications excluded mRS 0.

**Table 2:** The final study population of 120 patients alive at 3 years, 88 after Hunt & Hess scale 4 and 32 after Hunt & Hess scale 5 on admission. The distribution of clinical variables (rows) within each modified Rankin Scale group (mRS 0 – mRS 5 columns) is given in percentages and corresponding fractions. In addition, the division of mRS 0–5 is given for the H&H 4 row and H&H 5 row, as well as for the ICH (present) row

	Total alive at 3 years	Median mRS	mRS 0 (*n =* 17)	mRS 1 (*n =* 37)	mRS 2 (*n =* 20)	mRS 3 (*n =* 16)	mRS 4 (*n =* 23)	mRS 5 (*n =* 7)	*P* values
mRS at 3 years, % (*n*)	100% (120)	2	14% (17 / 120)	31% (37 / 120)	17% (20 / 120)	13% (16 / 120)	19% (23 / 120)	6% (7 / 120)	ref
Median age at aSAH, years (IQR)	52 (45–60)		50 (46–57)	54 (48–60)	49 (34–60)	53 (39–65)	52 (45–66)	54 (51–64)	ns
Females, % (*n*)	61% (73)	2	76% (13 / 17)	57% (21 / 37)	65% (13 / 20)	44% (7 / 16)	65% (15 / 23)	57% (4 / 7)	ns
H&H 4, % (*n*)	73% (88)	2	82% (14 / 17) 16% (14 / 88)	70% (26 / 37) 29% (26 / 88)	60% (12 / 20) 14% (12 / 88)	81% (13 / 16) 15% (13 / 88)	74% (17 / 23) 19% (17 / 88)	86% (6 / 7) 7% (6 / 88)	ns
H&H 5, % (*n*)	27% (32)	2	18% (3 / 17) 9% (3 / 32)	30% (11 / 37) 35% (11 / 32)	40% (8 / 20) 25% (8 / 32)	19% (3 / 16) 9% (3 / 32)	26% (6 / 23) 19% (6 / 32)	14% (1 / 7) 3% (1 / 32)	ns
ICH, % (*n*)	59% (71)	3	29% (5 / 17) 7% (5 / 71)	51% (19 / 37) 27% (19 / 71)	55% (11 / 20) 15% (11 / 71)	75% (12 / 16) 17% (12 / 71)	83% (19 / 23) 27% (19 / 71)	71% (5 / 7) 7% (5 / 71)	0.01
Median ICH volume, cm^3^ (IQR)	22 (11–56)		7 (3–17)	18 (9–28)	16 (4–55)	35 (16–45)	64 (42–101)	17 (4–48)	< 0.01
IVH, blood clot, % (*n*)	26% (31)	1	29% (5 / 17)	32% (12 / 37)	10% (2 / 20)	31% (5 / 16)	22% (5 / 23)	29% (2 / 7)	ns
IVH, blood sediment only, % (*n*)	49% (59)	2	59% (10 / 17)	43% (16 / 37)	60% (12 / 20)	38% (6 / 16)	43% (10 / 23)	71% (5 / 7)	ns
Shunted hydrocephalus, % (*n*)	46% (55)	2	35% (6 / 17)	51% (19 / 37)	40% (8 / 20)	44% (7 / 16)	48% (11 / 23)	57% (4 / 7)	ns
Epilepsy only after aSAH, % (*n*)	28% (33)	3	na*	22% (8 / 37)	30% (6 / 20)	38% (6 / 16)	48% (11 / 23)	29% (2 / 7)	ns
Depression only after aSAH, % (*n*)	28% (33)	3	na*	35% (13 / 37)	15% (3 / 20)	31% (5 / 16)	43% (10 / 23)	29% (2 / 7)	ns
Psychosis only after aSAH, % (*n*)	13% (16)	1	na*	27% (10 / 37)	5% (1 / 20)	13% (2 / 16)	13% (3 / 23)	0% (0 / 7)	ns
Drug treated hypertension, % (*n*)	66% (79)	1	76% (13 / 17)	73% (27 / 37)	50% (10 / 20)	63% (10 / 16)	65% (15 / 23)	57% (4 / 7)	ns
Drug treated diabetes type 2, % (*n*)	6% (7)	1	6% (1 / 17)	8% (3 / 37)	0% (0 / 20)	13% (2 / 16)	0% (0 / 23)	14% (1 / 7)	ns

Association of each variable to the modified Rankin Scale (0–5) using the Kruskal-Wallis Test. The five variables below the shunted hydrocephalus became known only during the follow up by the prescribed drug purchases

Abbreviations: *mRS*, modified Rankin Scale; *aSAH*, aneurysmal subarachnoid hemorrhage; *IQR*, 25% and 75% range; *H&H*, Hunt and Hess scale; *ICH*, intracerebral hematoma; *IVH*, intraventricular hematoma; *na*, not applicable; *ns*, not significant

*By definition drug treated epilepsy, depression and/or psychosis excluded mRS 0. Their p-value was calculated for mRS 1–5

### Literature review

Firstly, PubMed was searched in November 2020 for English articles on human aSAH published between 2010 and 2020 with the words (Aneurysm* AND “subarachnoid hemorrhage”) AND ((poor-grade) OR (poor grade)) AND (long-term AND (mortality OR survival OR outcome OR prognosis)) AND (12 months OR twelve-month). This gave 14 hits.

Secondly, we identified the original clinical aSAH patient cohorts, excluding case reports, duplicate publications, systematic reviews, and meta-analyses.

Thirdly, we approved the aSAH cohorts fulfilling the following criteria:

1. Poor condition on acute admission, expressed as H&H 4 or 5, or WFNS 4 or 5, or GCS 3 to 12.
2. Over 200 poor-grade aSAH patients to have a significant group of long-term survivors (mortality rate at 50%).
3. Median or mean follow-up time over 12 months.
4. Outcome expressed at more than 12 months using the modified Rankin Scale (mRS), or Glasgow Outcome Scale (GOS), or Extended Glasgow Outcome Scale (GOSE).

Finally, none of the articles fulfilled the criteria.

### Statistical methods

The categorical variables were expressed in proportions, and the *χ*2 test was used in comparisons. The continuous variables were expressed in medians, quartiles, and ranges, and the non-parametric tests were used in comparisons. The Kaplan-Meier analysis was used to calculate the cumulative mortality rates, and the log-rank test was used to test for differences between groups. Independent risk factors for the clinical condition at 3 years of the 120 patients were searched with logistic regression analysis. *P* values < 0.05 were considered significant. We used the SPSS 27 statistical software (SPSS, Inc., Chicago, IL).

## Results

### Basic study population of 269 aSAH patients in poor condition on admission

**Fig. 2:**
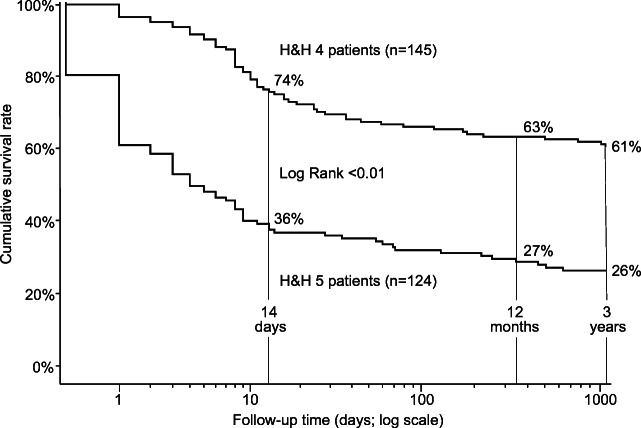
The cumulative survival rates for the H&H 4 (*n*=145) patients and the H&H 5 (*n*=124) patients after admission for acute aSAH. Cumulative survival rates at 14 days, 12 months, and 3 years of 269 consecutive aneurysmal subarachnoid hemorrhage (aSAH) patients admitted acutely in poor condition (H&H scale 4 or 5) to the Neurointensive Care Unit of the Kuopio University Hospital from its Eastern Finnish catchment population from 2005 to 2015. The follow-up time is logarithmic to emphasize the early high mortality. H&H, Hunt & Hess scale

A total of 269 consecutive aSAH patients were in poor condition (H&H 4, *n*=145; H&H 5, *n*=124) on admission to the KUH Neurointensive Care Unit (flowchart in Fig. 1, Table 1). Table 1 presents their clinical characteristics on admission, as well as those alive at 12 months and 3 years. Of the 269 patients, 155 (58%) were alive at 14 days, 125 (46%) at 12 months, and 120 (45%) at 3 years, significantly more often the H&H 4 patients (Fig. 2). Decompressive craniectomy (DC) was performed in 35 (13%) patients in a median of 2 (IQR 1–4) days; 13 (37%) of them died within 14 days (Table 1).

### Final study population of 120 aSAH patients alive at 3 years

**Fig. 3:**
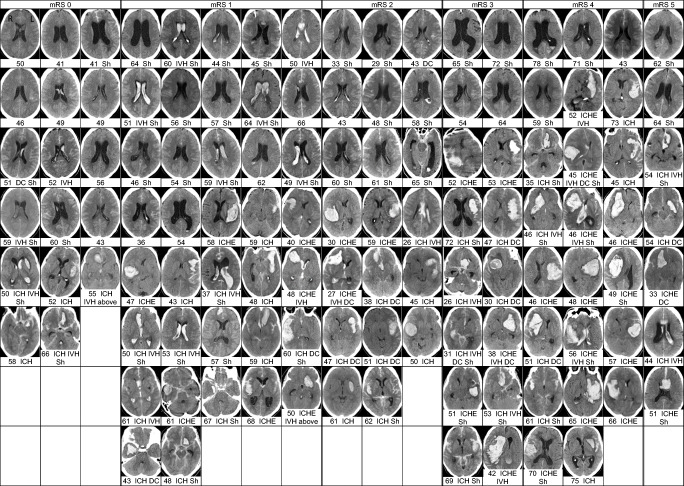
Primary CT scans of the 120 aSAH patients alive at 3 years, arranged according to their mRS. A total of 269 aSAH patients had been acutely admitted in poor condition (H&H 4; *n*=145 or H&H 5; *n*=124) to the neurointensive care between 2005 and 2015 from Eastern Finnish catchment population (Fig. 1). At 3 years, 120 (45%) of them were alive. This presents one representative CT slice of each patient (age in years) on admission, arranged according to the patient’s mRS from 0 to 5 at 3 years. There were 71 (59%) intracerebral hematomas (ICHs) on admission, 29 (41%) of them microsurgically evacuated (ICHE). There were 31 (26%) intraventricular hematomas (IVHs). Extraventricular drainage (EVD) was used in 105 (88%) patients and not used in 15 (13%). A total of 16 (13%) decompressive craniectomies (DC) were performed. In two patients in poor condition (mRS 5), cranioplasty was not performed. A ventriculoperitoneal shunt (Sh) was subsequently inserted in 55 (46%) patients. The collection is zoomable to observe details in different CT slices. By using the Find command, the following can be simultaneously identified in the whole collection: mRS, ICH, ICHE, IVH, DC, or Sh, or their different combinations. aSAH, aneurysmal subarachnoid hemorrhage; H&H, Hunt and Hess scale; CT, computed tomography; mRS, modified Rankin Scale; ICH, intracerebral hematoma; ICHE, intracerebral hematoma microsurgically evacuated; IVH, intraventricular hematoma (blood clot); EVD, extraventricular drainage; DC, decompressive craniectomy; Sh, shunt. Small blood sedimentations in occipital tips of the lateral ventricles were not considered as intraventricular clots (see above IVH)

At 3 years, 120 (45%) patients were alive, 88 (73%) after H&H 4 and 32 (27%) after H&H 5 on admission (Table 1). Their clinical condition at 3 years is distributed as follows (Table 2, Fig. 3): 14% mRS 0; 31% mRS 1; 17% mRS 2; 13% mRS 3; 19% mRS 4; and 6% mRS 5 (nursing home or hospital). By 3 years, 33 (28%) patients had started new anti-epileptic (AE) medication, 33 (28%) new anti-depressive (AD) medication, and 16 (13%) new anti-psychotic (AP) medication (Table 2). A total of 55 (46%) patients carried a ventriculoperitoneal shunt (Tables 1 and 2, Fig. 3).

### Primary CT scans of 120 aSAH patients and their mRS at 3 years

Figure 3 presents one representative CT slice of each patient, together with the surgical interventions (see below), arranged according to the patient’s modified Rankin Scale (mRS) from 0 to 5 at 3 years. There were 71 (59%) intracerebral hematomas (ICH) with a median volume of 22 cm^3^ (IQR 11–56), 47 (66%) from the middle cerebral artery (MCA) sIA, and 12 (17%) from the anterior communicating artery (AComA) sIA. There were 31 (26%) intraventricular blood clots (IVH), 15 (48%) from the AComA sIA, and 10 (32%) from the MCA sIA. Mere intraventricular blood sediments were seen in 59 (49%). Acute hydrocephalus was present in 76 (63%) patients.

### Neurointensive care and surgical interventions of 120 aSAH patients

Extraventricular drainage (EVD) was started in 105 patients (88%). Table 3 cross-tabulates the 12 combinations of microsurgical evacuation of ICH (*n*=29), microsurgical clipping (*n*=55) or endovascular occlusion (*n*=64) of ruptured sIA, and decompressive craniectomy (*n*=16). In each of the 12 patient groups, median mRS at 3 years is presented. Figure 3 shows the representative CT slices for each of the 12 patient groups. Intraventricular blood clots, present in 31 (26%) patients (Fig. 3), were not evacuated microsurgically or endoscopically; one patient had intraventricular alteplase (t-PA) thrombolysis.

**Table 3:**
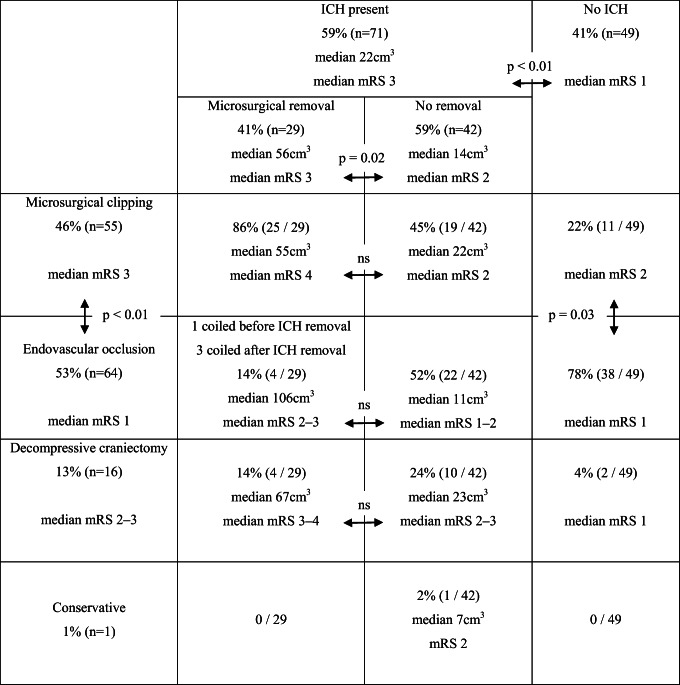
The final study population of 120 patients alive at 3 years. The patients are cross-tabulated into 12 groups according to the combinations of the surgical interventions. For each patient group, median modified Rankin Scale at 3 years is presented. Figure 3 shows the representative computed tomography slices for each of the 12 patient groups

*ICH*, intracerebral hematoma; *mRS*, modified Rankin Scale

Double-headed arrows denote the comparisons between two groups with mRS as the end variable, using the Mann-Whitney *U* test

### Shunt dependency among 120 aSAH patients

A total of 55 (46%) patients had a ventriculoperitoneal shunt (Fig. 3), inserted in a median of 17 days (IQRs 9 and 64) after aSAH. In the 55 shunted patients, the ventricles on admission contained IVH clot in 18 (33%) (Fig. 3), mere blood sedimentation in 34 (62%), and no blood in 3 (5%). Until 3 years, 32 (58%) patients had no shunt revisions (median mRS 1). A total of 23 (42%) patients had shunt revisions (median mRS 3), 11 once, and 12 two to five times. At 3 years, the 65 non-shunted patients had a median mRS of 2.

### Cranioplasty after decompressive craniectomy among 120 aSAH patients

DC was performed in 16 (13%) patients (Table 1 and 3, Fig. 3) in a median of 2 (IQR 0–5) days after admission. In 14 of the 16 DC patients, cranioplasty was performed in a median of 94 (IQR 46–127) days after DC, always with the own frozen bone flap; 4 flaps were later replaced with an artificial flap.

### Favorable condition (mRS 0 or 1)

At 3 years, 54 patients (median 55 years) had mRS 0 or 1, 45% of the 120 three-year survivors but only 20% of all the 269 original aSAH patients with H&H 4–5 on admission (Table 2). As many as 17 (31%) patients had no symptoms (mRS 0; no AE, AD, or AP drug use) despite minor ICH in five, IVH blood clot in five, or shunt in six (Table 2, Fig. 3).

### Unfavorable condition (mRS 4 or 5)

At 3 years, 30 patients (median 57 years) had mRS 4 or 5, 25% of the 120 three-year survivors but only 11% of the 269 original H&H 4–5 aSAH patients (Table 2). As many as 24 (80%) patients had ICH (14 evacuated) while only 6 had no ICH at all, 7 (23%) had an IVH clot, and 15 (50%) had a shunt (Tables 2 and 3, Fig. 3). In multivariate testing of available data, the ICH volume stood out as a significant predictor of mRS at 3 years.

## Discussion

### Long-term survivors among “poor-grade” aSAH patients

After acute aSAH, “poor condition” or “poor grade” on admission to the first hospital for CT and after transfer to the neurointensive care predicts early mortality so high that aSAH patients brain dead within 14 days are a significant group of organ donors [21, 37, 45]. The acute effect may be so devastating that some aSAH patients do not reach alive the first hospital and diagnostic CT [30]. The long-term mortality, often presented at 12 months [47], is also high: 37% in H&H 4 and 73% in H&H 5 in our present study (Fig. 2). A minority of H&H 4–5 or WFNS 4–5 patients will become true long-term survivors, in the present study 120 (45%) patients alive at 3 years (Table 2), albeit being stroke-risk carriers and prone to further vascular events [16]. They have remained an unrecognized group among stroke survivors for obvious reasons: prospective databases with long recruitment and outpatient follow-up periods would be virtually impossible to maintain, while much of literature is focused on “aneurysm treatment” [43]. We have retrospectively analyzed their clinical condition during very long follow-ups: excess mortality (median 12 years) [17]; epilepsy (median 6 years) [18]; anti-depressants (median 9 years) [19]; anti-psychotics (median 9 years) [39]; and shunt dependency (median 8 years) [1].

### Visualization of a complex dataset with clinical lifelines

In the present study, we constructed the clinical date point calendaric lifelines for the 269 consecutive aSAH patients in poor condition (54% with H&H 4 and 46% with H&H 5) on admission to the Neurointensive Care Unit of the Kuopio University Hospital (KUH) from the defined KUH catchment population from 2005 to 2015 (Fig. 1, Table 1). For the lifeline analysis, each patients’ medical data, using the Finnish personal identification code, was obtained from the nationwide registries, and fused into the Kuopio Intracranial Aneurysm Patient and Family Database. The cross-sectional study population consisted the 120 consecutive patients alive at 3 years, 73% with H&H 4 and 27% with H&H 5 on admission (Table 1, Table 2). We found it impossible, using sentences and risk factors only, to present a comprehensive view of the associations of multiple intertwining factors during the neurointensive care vs. the clinical conditions at 3 years. For the same reason, the patients who deteriorated from H&H 1–3 to H&H 4–5 during the neurointensive care were excluded. For visual estimation and browsing, one representative slice of each of the 120 primary CT scans (ICH; IVH; hydrocephalus) and the interventions (extraventricular drainage; sIA occlusion; ICH evacuation; decompressive craniectomy; shunt) were compiled together, according to the mRS (0 to 5) at 3 years (Fig. 3) (see also 45 asymptomatic meningiomas by Näslund et al. in 2020 in *Acta Neurochirurgica* [35]). Furthermore, the interventions and the presence or absence of ICH were cross-tabulated into 12 groups (Table 3).

### Aneurysmal intracerebral hemorrhage and the brain connectome

Of the 120 H&H 4 or 5 patients alive at 3 years, 71 (59%) had an intracerebral hematoma (ICH) with a median volume of 22 cm^3^, mostly (47; 66%) from a middle cerebral artery (MCA) sIA, and (12; 17%) from an anterior communicating artery (AComA) sIA. Aneurysmal ICH, depending on its volume and location, is the single most damaging and deadly complication of acute aSAH [51]. In the present study, ICH volume was a significant predictor of mRS at 3 years (Table 2). Arterial blood yet from the ruptured aneurysm wall tears and enters the adjacent brain tissue, forming a permanent brain cavity, filled with ICH clot. MRI (diffusion/perfusion/tractography) may give further prognostic information on brain edema [42], early brain ischemia [11, 14], and injury to white matter tracts and nuclei, e.g., in relation to motor function [4] or impaired consciousness [20]. The local injury of the brain connectome is permanent; i.e., no axonal connections will redevelop across the ICH cavity that later in neuroimaging appears as a CSF cavity. Convalescence, e.g., from acute hemiparesis or aphasic disorder, depends on the functional re-organization of the connectome around the cavity.

### Acute evacuation of aneurysmal ICH

In our series, 71 (59%) of the 120 patients presented with ICH, and 29 ICHs (median volume 56 cm^3^) were microsurgically evacuated. Acute evacuation relieves expansion and pressure against the cavity wall, but the extent of brain tear remains unchanged. Only gentle subtotal evacuation is realistic as aneurysmal ICHs are rooted locally among cortical and perforating arteries [49]. Nevertheless, the ICH clot inducing neuroinflammation becomes smaller [53]. However, there is not yet concrete evidence on long-term benefits of acute aneurysmal ICH evacuation [14]. Notably, in spontaneous ICHs and IVHs, there is increasing interest in acute endoscopic and stereotactic evacuation [3, 34].

### Decompressive craniectomy and cranioplasty

In our series, DC was performed in 35 (13%) of the 269 aSAH patients with H&H 4 or 5 on admission: 16 of them (46%) were among the 120 three-year survivors. After this series, we abandoned the frozen bone flaps for artificial ones, because 19% of own bone flaps were removed due to complications in a Finnish study [24]. In a systematic review of 407 aSAH patients with WFNS 4 or 5, the effect of DC on functional outcome remained unknown because of the lack of robust control groups [2].

### Aneurysmal intraventricular hemorrhage

Aneurysmal IVHs of various volumes are frequent in acute aSAH patients [38, 44]. In our basic series, IVHs ranged from mere blood sediments (38%) in the occipital horns to clots (34%), some casting one or both lateral ventricles. IVH clots may co-exist with adjacent ICH, in 64 (24%) cases in our series, adding instant brain tissue injury to multiple potentially harmful effects of IVH. With pathobiology poorly understood, they include neuroinflammation [10]; alteration in ependymal cells, ciliary beat, arachnoid villi, CSF production and resorption, and glymphatic circulation; and clinically manifest hydrocephalus (acute; subacute; latent). IVH with enlarged ventricles often require prolonged EVD with ICP and CSF outflow monitoring, with the risk of catheter occlusions and exchanges, and eventual bacterial meningitis [1]. In principle, it would make sense to reduce clots and casts in the lateral ventricles, even III and IV, with neuroendoscopy as soon as feasible. In spontaneous IVH, recent meta-analysis suggested that endoscopy improved the survival and prognosis with the lowest rate of shunts and infection [34].

### Secondary hydrocephalus and shunt dependency

Secondary hydrocephalus is frequent among aSAH survivors, and several risk scores have been published [1]. The pathobiology is poorly understood but neuroinflammation is a candidate [10], activating in the acute phase and possibly exerting a long-term dysfunction in the CSF environment, with tendency of valves and catheters to occlude, as well as shunt infection. EVD is an instant indicator of shunt dependency, and concomitant bacterial meningitis or ventriculitis increases the risk [1]. Surprisingly, in our series and other cohorts, even normal ventricle size and sediments of IVH only could be followed by latent shunt dependency [1, 8, 52]. The long-term outcome and quality of life of shunted aSAH survivors is virtually unknown according to our literature review. Long-term follow-up studies will show whether shunt-dependent post-aSAH hydrocephalus is a degenerative brain disease, such as idiopathic normal pressure hydrocephalus (iNPH) after temporary improvement with a shunt [23].

### Outcome algorithms for H&H 4 or 5 aSAH patients on admission

Our pilot study, aimed to analyze a significant group of long-term aSAH survivors, did not include enough data (e.g., previous conditions of patients; ICP monitoring; EVD outflow; acute or delayed ischemic brain injury; electrolyte disturbances; cardiopulmonary complications; CNS or systemic infections; and complications of management) for computerized (e.g., machine learning) prediction on admission or during neurointensive care for subsequent mortality and outcome. Still, some aspects concerning triage on admission can be extracted from the 120 H&H 4–5 survivors at 3 years, as visualized in Fig. 3 and Table 3.

Firstly, in 49 patients (41%) of 120 patients with H&H 4 or 5 on admission, the primary CT scan did not show immediate brain injury by ICH. Their mRS range at 3 years from 0 to 5 indicates that coma and extension to pain are unreliable triage predictors of dismal outcome [28], interfered, e.g., by seizures, hydrocephalus, brain herniation, sedation, intubation, and ventilator care. Acute MRI would add information on possible acute ischemic brain injury [14].

Secondly, large ICHs in eventual mRS 3 to 5 survivors (maximum 200 cm^3^ in our series) may question chances of survival at the initial triage for neurointensive care. Large ICHs raise the question whether lives saved with neurointensive care are “worth living” with mRS 4 to 5 in the long run, i.e., whether such logistics would be ethically acceptable in neuroacutology. With the opt-out system for brain death and organ donation in Finland since 2010, we also admit “hopeless” aSAH patients, provided that the National Transplantation Service does not by phone exclude the possibility of organ donation due to concomitant diseases or conditions [21, 45]. The donors of kidneys included brain dead aSAH patients even at their 80’s. In Finland, the annual costs of dialysis (about 40,000 euros) far exceed the costs after the first year of a kidney transplant patient [15]. Eventually, evaluating CT scans in Fig. 3, the lives saved with large ICH and dismal (“unacceptable”) long-term condition (mRS 4 or 5) were few: 23 (9%) of the 269 H&H 4 or 5 patients on admission (Fig. 3).

Thirdly, it was surprising, e.g., how the six mRS 0 or 1 patients (median 58 years) with ICH over 27 cm^3^ (the largest quartile) managed to recover so well at 3 years (Tables 1 and 2, Fig. 3). This unexpected *resilience to brain injury* requires further investigations.

### Strengths and limitations of the study

The strengths derive from the free tax-paid Finnish health care system, as well as the automatic archival of clinical data, using the Finnish identity codes, in the national clinical registries. Finland is divided into exclusive catchment areas among the 5 university hospitals which allows the creation of disease cohorts that are minimally selected and biased. The Kuopio Intracranial Aneurysm Patient and Family Database reliably reflects aSAH in the Eastern Finnish population and allows reconstruction of the clinical date point lifelines of all diagnosed aSAH patients, also using data from the national clinical registries. Our study is retrospective although the database prospectively collected all aSAH patients through the study period.

There are also limitations. The evaluation of the clinical condition of the 120 three-year survivors was based on the available case reports and the data available in the national registries. In this pilot study, the patients who deteriorated from H&H 1–3 on admission to H&H 4–5 later were excluded from the final study population. Finally, the extensive digital neurointensive care monitoring data, available in the national database, was not used [41]; such data are indispensable in further studies with machine learning algorithms.

### Suggested further research

Firstly, retrospective re-reconstruction of clinical lifelines for individual aSAH patients, or any neuroacutology patients, is arduous patchwork at present, for clinical research or quality control. The IT systems of the hospital catchment area should automatically provide individual digital timelines (in minute scale) from the first contact through all phases to, e.g., the neurointensive care. Such quality control practice would aid to detect and prevent delays and deviations in aSAH logistics that might risk the final outcome, including time of intubation or release of tentorial herniation.

Secondly, the long-term course of consequences of acute aSAH on the brain has not been adequately studied. Long-term understanding would require consecutive MRI scans (diffusion/perfusion and tractography), at acute phase (acute hemorrhagic injuries, severing of tracts, hydrocephalus, and ischemia), at subacute and late subacute phases, at 12 months (permanent hemorrhagic and ischemic injuries), and after a few years (neurodegeneration).

Thirdly, neuroinflammation might function as an umbrella for cellular and molecular biology research of several calamities of the aSAH brain: early brain ischemia; ICH; IVH; ischemic events during occlusive IA therapy; delayed ischemia; shunt-dependent hydrocephalus—also postulated neurodegeneration in the post-aSAH brain [5].

### Clinical conclusions

1. Poor clinical condition (H&H 4–5, WFNS 4–5) on admission does not reliably predict the long-term outcome, particularly so if the patient survives the early excess mortality (14 days and 12 months) caused by aSAH. The risk of ending in poor long-term condition is relatively small, e.g., 8.6% at mRS 4 and 2.6% at mRS 5 (hospice care) at 3 years in our series.
2. We propose that virtually all acute aneurysmal subarachnoid hemorrhage (aSAH) patients in poor condition on admission are immediately transferred to neurointensive care. In the countries with national presumed consent (opt-out), aSAH patients with dismal prognosis can be admitted to neurointensive care as potential organ donors, also at high age.
3. Better understanding of the consequences of acute aSAH on the brain would require consecutive MRI scans (diffusion/perfusion and tractography), at acute phase (acute hemorrhagic injuries, severing of tracts, hydrocephalus, and ischemia), at subacute and late subacute phases, at 12 months (permanent hemorrhagic and ischemic injuries), and after a few years (neurodegeneration).

## Abbreviations

ACA: Anterior cerebral artery and peripheral segments
AComA: Anterior communicating artery
aSAH: Aneurysmal subarachnoid hemorrhage
BA: Basilar artery trunk and bifurcation
CNS: Central nervous system
CT: Computed tomography
DC: Decompressive craniectomy
EVD: Extraventricular drainage
H&H: Hunt and Hess scale
ICA: Internal carotid artery trunk and bifurcation
ICH: Intracerebral hematoma
ICHE: Intracerebral hematoma microsurgically evacuated
ICP: Intracranial pressure
IQR: 25% and 75% range
IVH: Intraventricular hematoma (blood clot)
KUH: Kuopio University Hospital
MCA: Middle cerebral artery and peripheral segments
mRS: Modified Rankin Scale
PICA: Posterior inferior cerebellar artery
Sh: Shunt
sIA: Saccular intracranial aneurysm
WFNS: World Federation of Neurosurgical Societies
